# Creating Physical 3D Stereolithograph Models of Brain and Skull

**DOI:** 10.1371/journal.pone.0001119

**Published:** 2007-10-31

**Authors:** Daniel J. Kelley, Mohammed Farhoud, M. Elizabeth Meyerand, David L. Nelson, Lincoln F. Ramirez, Robert J. Dempsey, Alan J. Wolf, Andrew L. Alexander, Richard J. Davidson

**Affiliations:** 1 Waisman Laboratory for Brain Imaging and Behavior, Waisman Center, Madison, Wisconsin, United States of America; 2 Medical Scientist Training Program, University of Wisconsin School of Medicine and Public Health, University of Wisconsin, Madison, Wisconsin, United States of America; 3 Neuroscience Training Program, University of Wisconsin School of Medicine and Public Health, University of Wisconsin, Madison, Wisconsin, United States of America; 4 Howard Hughes Medical Institute (HHMI) Teaching Fellows Program, Wisconsin Program for Scientific Teaching, Department of Bacteriology, University of Wisconsin, Madison, Wisconsin, United States of America; 5 Paul P. Carbone Comprehensive Cancer Center, University of Wisconsin, Madison, Wisconsin, United States of America; 6 Biology New Media Center, University of Wisconsin Biotechnology Center, Madison, Wisconsin, United States of America; 7 Department of Medical Physics, University of Wisconsin, Madison, Wisconsin, United States of America; 8 Department of Biochemistry, College of Agricultural and Life Science, University of Wisconsin, Madison, Wisconsin, United States of America; 9 Department of Neurological Surgery, University of Wisconsin, Madison, Wisconsin, United States of America; Center for Genomic Regulation, Spain

## Abstract

The human brain and skull are three dimensional (3D) anatomical structures with complex surfaces. However, medical images are often two dimensional (2D) and provide incomplete visualization of structural morphology. To overcome this loss in dimension, we developed and validated a freely available, semi-automated pathway to build 3D virtual reality (VR) and hand-held, stereolithograph models. To evaluate whether surface visualization in 3D was more informative than in 2D, undergraduate students (n = 50) used the Gillespie scale to rate 3D VR and physical models of both a living patient-volunteer's brain and the skull of Phineas Gage, a historically famous railroad worker whose misfortune with a projectile tamping iron provided the first evidence of a structure-function relationship in brain. Using our processing pathway, we successfully fabricated human brain and skull replicas and validated that the stereolithograph model preserved the scale of the VR model. Based on the Gillespie ratings, students indicated that the biological utility and quality of visual information at the surface of VR and stereolithograph models were greater than the 2D images from which they were derived. The method we developed is useful to create VR and stereolithograph 3D models from medical images and can be used to model hard or soft tissue in living or preserved specimens. Compared to 2D images, VR and stereolithograph models provide an extra dimension that enhances both the quality of visual information and utility of surface visualization in neuroscience and medicine.

## Introduction

Leonardo da Vinci was the first to model brain structure by injecting molten wax into the ventricle of an oxen brain [1]. Since then, neuroanatomical models have shown utility in neuroscience and medicine in areas such as education, diagnosis, and surgical planning [2], [3]. Currently, classical modelling techniques are being supplanted by modern methods that emphasize three-dimensional anatomical relationships using imaging techniques [4], [5]. In this report, we describe and evaluate a modern method to replicate an individual's anatomy as a physical, hand-held model by using reverse engineering and rapid prototyping stereolithography [6].

Reverse engineering is a process in which a 3D physical object is scanned using an MRI or CT, for example, and the images are used to render a three-dimensional virtual model. Rapid prototyping is a new technology that uses this virtual model to print or fabricate a physical model. Stereolithography is a rapid prototyping technique with several variants; and, in our application, layers of plaster can form a solid, physical model after a binding agent is applied to each printed layer.

Although other hand-made and computerized modelling approaches have been used to model neuroanatomy [7], [8], rapidly prototyped models preserve anatomical scale and anatomical relationships, are three-dimensional, are understood by visual and tactile learners, do not require software training, and can be produced to the anatomical specifications of the individual patient. Compared to computerized virtual models which have the capability to virtually dissect an object in an infinite number of ways, physical models can be intuitively held and rotated, can be interactively manipulated regardless of complexity, and are accessible without the need for computers or advanced training.

In this report, we developed a streamlined procedure to replicate human brain and skull morphology using freely available software to produce CT/MRI-assisted reverse engineered VR and stereolithograph models; validated the scaling precision of our modelling pathway for quality control; and evaluated 2D images, virtual models (VM), and stereolithograph models (SM) of brain and skull for the quality of visual information and utility of these models.

## Analysis

### Algorithm

**Figure 1:**
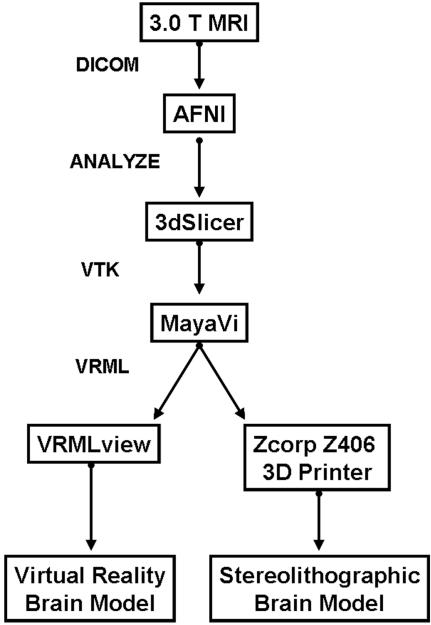
MRI-based Reverse Engineering Processing Pathway. Freely available software and file formats used to produce virtual and stereolithograph brain models are diagrammed. AFNI is available from [http://afni.nimh.nih.gov/]; 3dSlicer is available from [http://www.slicer.org/]; MayaVi is available from [http://mayavi.sourceforge.net/].

**Figure 2:**
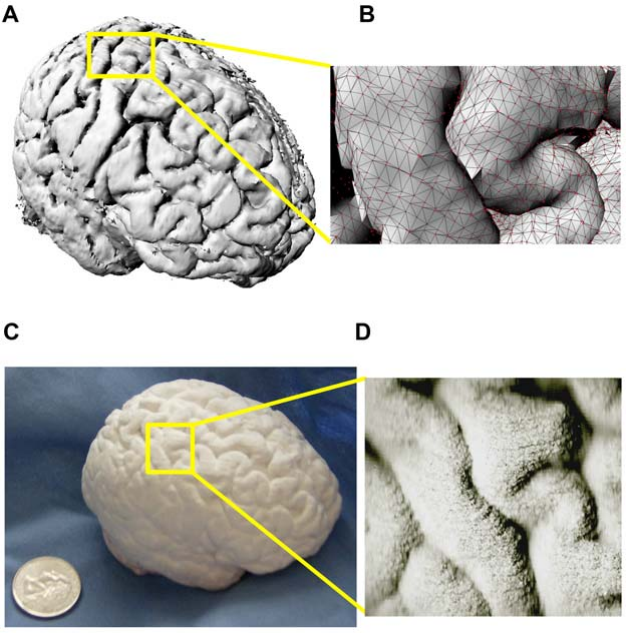
VR and Rapidly Prototyped Stereolithographic Human Brain Model. The processing pathway we developed can replicate living specimens. (a) Cortical isosurface of virtual whole human brain. (b) Right hemisphere cortical isosurface of pre- and postcentral (left) gyrus with wireframe mesh (black) and vertices (red). (c) Physical whole human brain stereolithograph replica. (d) Right hemisphere pre- and postcentral (left) gyrus for comparison with Figure 2b.

We developed an open-source processing pathway to visualize VR and stereolithograph models (Figure 1). With University of Wisconsin-Madison IRB approval, reverse engineering of brain began with 3D SPGR MRI acquisition of living human brain images on a 3.0 Tesla GE Signa (General Electric Medical Systems; Waukesha, WI) scanner (114 axial slices 1.3 mm thick; 256×256 resolution; pixel size 0.9375×0.9375). DICOM images were loaded into the NIH Analysis of Functional Neuroimaging (AFNI) software package [9] for automated inhomogeneity correction and brain isolation through skull stripping. Although a mesh is commonly used to model the brain's surface (as in Figure 2b), this method failed to print because the mesh lacks information about the thickness of the surface and is too thin to print. For this reason, we preserved the volume dimension of this dataset by generating an isosurface which preserves surface morphology and has enough thickness to be printed. After exporting these files in an ANALYZE format, these images were reconstructed in three dimensions and saved in the visualization tool kit (.vtk) format using 3dSlicer [10]. The reconstructed images were converted to the virtual reality markup language (VRML) for three dimensional printing using isosurface visualization without scalar coloring in MayaVi [11]. We viewed and navigated the VRML brain model with VRMLView (Systems in Motion; Norway). The virtual whole brain isosurface (Figure 2a) and cortical mesh clearly delineated sulci from gyri (Figure 2b).

### Validation

We fabricated an intact, half scale, whole brain 3D stereolithograph model (SM) which preserved cortical morphology using the VRML model to drive the 3D Printing (3DP) [6] process on a Zcorp Z406 3D printer (Z Corporation; MA, USA) at the UW-Madison New Media Center. The whole brain 3D stereolithograph model (Figure 2c) preserved the cortical morphology (Figure 2d; compare to Figure 2b) of the virtual reality markup language (VRML) model.

Maximum length (mm) measurements along the primary right-left (RL), anterior-posterior (AP), and superior-inferior (SI) axes were respectively taken for the VM and SM models with AFNI using DICOM images and with ImageJ [12] using images collected with an HP (Hewlett Packard, USA) PSC 1210 scanner. The primary axes length measures for the scaled stereolithograph model (RL,AP,SI: 67.2, 90.1, 63.2 mm) were as expected based on the virtual brain model (RL,AP,SI: 134.1, 179.1, 133.9 mm). The SM brain was reduced 3.75 mm (5.9%) in the SI dimension during model construction because the model was printed in layers from the inferior to superior direction and the weight of the model caused compression along this dimension. Temperature, humidity, and seasonal calibration also contributed to error in the SI dimension. The printer operator can correct this printing artifact by adjusting the length of the model in the dimension of model growth.

**Figure 3:**
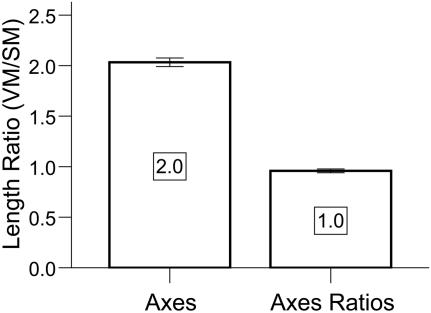
Validation of Processing Pathway. The stereolithographic model preserved the VM:SM average axes scaling factor of 2:1 [Mean+/−SEM = 2.03+/−0.04; t(2) = 0.79;p = 0.51, 2-tailed] and the 1:1 average axes proportion [Mean+/−SEM = 0.96+/−0.02; t(2) = −2.2;p = 0.16, 2-tailed]. Error bars are SEM.

For validation, we tested whether the average virtual to stereolithograph ratios for the primary axes lengths 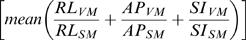 preserved the 2:1 VM:SM axis scaling factor and the stereolithograph model average axisproportions 
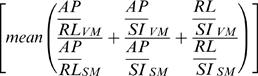
 preserved the 1:1 VM:SM ratio. These measures indicated that our half-scale stereolithograph brain model preserved the dimensions of the virtual model (Figure 3).

**Figure 4:**
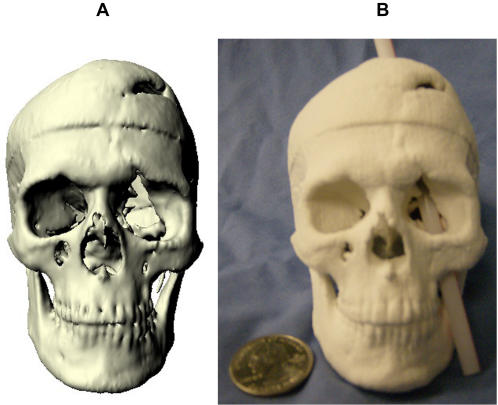
Stereolithograph of Phineas Gage's Skull. The processing pathway we developed can replicate preserved specimens. (a) Virtual model of Phineas Gage's skull. (b) With this physical 3D model of Phineas Gage's skull, we illustrate the approximate path of the tamping iron that produced Phineas Gage's famous injury.

We then applied our method to produce a VRML (Figure 4a) and physical 3D model (Figure 4b) of Phineas Gage's skull. As the raw CT scans of Phineas Gage's skull were not available because scan requests from prior authors failed, we produced the 3D model from a video clip of multi-plane, computed tomography (CT) images taken of Phineas Gage's skull in 2004 [13]. The accurate physical reproduction of Phineas' skull required a virtual model with the correct dimensions; however, voxel size was not reported with the video clip [13] and a prior publication did not report measurements taken of the authentic Phineas skull [14]. Accurate replication of the virtual model was possible using a voxel size (RL,AP,SI: 0.48,0.8,0.8 mm) in which the VR skull dimensions matched the anatomical dimension measures of the authentic Phineas Gage skull (Table 1) provided by Dominic Hall, Curator of the Warren Anatomical Museum at Harvard Medical School which houses the authentic skull of Phineas Gage. The mpeg video clip was brought into AFNI, cropped to isolate sagittal images (RL,AP,SI: 458,352,342 voxels), exported in ANALYZE format, entered into our processing pathway (Figure 1), and volume rendered in Video S1. Caliper measurements (12″ digital caliper; Neiko Tools, USA) indicated that the half-scaled dimensions of the stereolithograph skull model were as expected (RL,AP,SI: 66.0,90.6,56.6 mm) and the printer operator successfully compensated for compression in the RL print out dimension. The stereolithograph skull model preserved the VM:SM average axes scaling factor of 2:1 [Mean+/−SEM = 1.99+/−0.00; t(2) = −1.73;p = 0.23, 2-tailed] and the 1:1 average axes proportion [Mean+/−SEM = 1.0+/−0.00; t(2) = −0.32;p = 0.78, 2-tailed].

**Table 1:** Dimension Estimates of Authentic Phineas Gage Skull.

Primary Axis Dimension	Anatomical Landmarks	Length Estimates (mm)
Right:Left (RL)	Right Extrema to Left Extrema	131
Anterior:Posterior (AP)	Glabella to Posterior Extrema	180
Superior:Inferior (SI)	Nasion to Gnathion	113

### Evaluation

Students enrolled in the University of Wisconsin-Madison course entitled “Ways of Knowing Biology,” voluntarily and anonymously evaluated the quality of visual information contained in the 2D, VRML, and stereolithograph models of brain and Phineas Gage's skull. We used the Gillespie rating scale (Table S1) to assess students' perception of biological image models relative to a baseline model [3]. In our assessment, 2D images were used as the baseline for assessing VRML and stereolithograph models for the quality of visual information and biological utility. Gillespie ratings were coded with 1 = Inferior, 2 = Similar/Equivalent, 3 = Superior (similar information more rapidly assimilated), 4 = Superior (additional information provided). Since all models were derived from the same data, differences in perceived visual information were a function of model type.

The Gillespie rating survey indicated that the 3D models were superior in surface visualization quality (Table 2) compared to 2D images (Gillespie rating = 2) for the brain VRML [t(49) = 37.5; p = 0.00], brain stereolithograph [t(49) = 12.7; p = 0.00], Phineas VRML [t(49) = 20.8;p = 0.00], and the Phineas stereolithograph models [t(49) = 14.1; p = 0.00]. The Wilcoxon signed ranks test indicated that the quality of visualization using VRML is superior to stereolithograph models for brain [Z = −4.88; p = 0.00; n = 50] and skull [Z = −2.29; p = 0.022; n = 50] and that biological image utility was greater for VRML than 2D models [Z = −6.13; p = 0.00; n = 50], was greater for stereolithograph than 2D models [Z = −4.97; p = 0.00; n = 50], and was greater for VRML than stereolithograph models [Z = −3.20; p = 0.001; n = 50].

**Table 2:** Gillespie Ratings of Visual Information and Biological Utility.

Question	Topic	Mean	SD	1	2	3	4
**VI**	**Quality of Visual Information, VI**						
1	Brain VRML	3.86	0.35	0	0	7	43
2	Brain Stereolithograph	3.10	0.61	1	4	34	11
3	Phineas VRML	3.58	0.54	0	1	19	30
4	Phineas Stereolithograph	3.28	0.64	0	5	26	19
**BU**	**Biological Utility, BU**						
1	2D	2.20	0.70	6	30	12	2
2	VRML	3.74	0.56	0	3	7	40
3	Stereolithograph	3.18	0.80	2	6	23	19

Questionnaire results reporting mean, standard deviation, and distribution of responses in each Likert-scaled category.

## Discussion

The reverse engineering and rapid prototyping pathway we developed has medical applications in biomodel guided stereotactic surgery [15], cranioplasty [16], aneurysm research and repair [17], and craniofacial reconstruction [18]. In neuroscience, this pathway has applications in producing phantoms of living or preserved specimens and in both basic science and imaging education. Even though the 3D models were derived from the same data as 2D images, 3D models display anatomical relationships in an extra dimension to enhance the quality of visual information and model utility compared to 2D images.

Efficient replication of the intact whole brain with high resolution was previously unattainable using rapid prototyping techniques due to limitations that have largely been overcome through advances in neuroimaging software, computer hardware, and higher field magnets. Disadvantages that remain for rapid prototyping include monetary cost (approximately 50 cents/cc, excluding the cost of MR imaging and data post-processing) and time to print (approximately 2.5 vertical cm/hour). Our half-scaled 219 cc physical brain model cost approximately $110 and took 2.5 hours to print. The 1756 cc full brain would have cost about $880 and taken 5.5 hours to print. The cost would have been significantly more using other rapid prototyping technologies.

We developed and validated a freely available method to model brain and skull anatomy in three dimensions using images collected with multiple modalities. Virtual reality and stereolithograph models are advantageous for observing surface morphology compared to two-dimensional planar images and can be manipulated using a data glove or bare hand, respectively. A disadvantage of the stereolithography approach compared to the VR approach is the lack of subcortical visualization and the inability to visualize the interior skull regions. Future adaptations of this technique should take advantage of 3D tissue printers which are currently being developed and the current color printing capability of 3D printers to delineate brain and skull regions.

## Supporting Information

Video S1 Volume Rendering of Phineas Gage's Skull
(0.77 MB MOV)

Table S1 Gillespie Rating Survey
(0.04 MB DOC)
