# Supplementary material for: Creating Physical 3D Stereolithograph Models of Brain and Skull
Source: PLoS One. 2007 Oct 31;2(10):e1119. doi: 10.1371/journal.pone.0001119 (PMC2040197; doi:10.1371/journal.pone.0001119)
Supplement: Table S1 — Gillespie Rating Survey (0.04 MB DOC) [file pone.0001119.s002.doc]

**Table S1: Gillespie Rating Survey**

Rate the quality of visual information contained in the following models.

| **Model** | **Inferior** | **Similar/**  **Equivalent** | **Superior**  **(similar information more rapidly assimilated)** | **Superior**  **(additional information provided)** |
| --- | --- | --- | --- | --- |
| **Brain Cortical Surface** | | | | |
| 2D | *Baseline* | | | |
| VRML |  |  |  |  |
| Stereolithograph |  |  |  |  |
|  |  | | | |
| **Phineas Gage Skull Injury** | | | | |
| 2D | *Baseline* | | | |
| VRML |  |  |  |  |
| Stereolithograph |  |  |  |  |

Evaluate the usefulness of different model types for understanding biology:

|  | **Not at all** | **A little** | **Somewhat** | **A lot** | **A great deal** |
| --- | --- | --- | --- | --- | --- |
| 2D |  |  |  |  |  |
| VRML |  |  |  |  |  |
| Stereolithograph |  |  |  |  |  |
